# SERS-Active Cu Nanoparticles on Carbon Nitride Support Fabricated Using Pulsed Laser Ablation

**DOI:** 10.3390/nano9091223

**Published:** 2019-08-29

**Authors:** Hossein Dizajghorbani-Aghdam, Thomas S. Miller, Rasoul Malekfar, Paul F. McMillan

**Affiliations:** 1 Atomic and Molecular Group, Physics Department, Faculty of Basic Sciences, Tarbiat Modares University, Tehran 14115-175, Iran; 2 Electrochemical Innovation Lab, Department of Chemical Engineering, University College London, Torrington Place, London WC1E 7JE, UK; 3 Department of Chemistry, Christopher Ingold Laboratories, University College London, 20 Gordon Street, London WC1H 0AJ, UK

**Keywords:** pulsed laser ablation, acetonitrile (CH_3_CN), Cu/gCN hybrids, localized surface plasmon resonance (LSPR), surface enhanced Raman scattering (SERS), surface enhanced resonance Raman scattering (SERRS)

## Abstract

We report a single-step route to co-deposit Cu nanoparticles with a graphitic carbon nitride (gCN) support using nanosecond Ce:Nd:YAG pulsed laser ablation from a Cu metal target coated using acetonitrile (CH_3_CN). The resulting Cu/gCN hybrids showed strong optical absorption in the visible to near-IR range and exhibited surface-enhanced Raman or resonance Raman scattering (SERS or SERRS) enhancement for crystal violet (CV), methylene blue (MB), and rhodamine 6G (R6G) used as probe analyte molecules adsorbed on the surface. We have characterized the Cu nanoparticles and the nature of the gCN support materials using a range of spectroscopic, structural, and compositional analysis techniques.

## 1. Introduction

Surface-enhanced Raman spectroscopy (SERS) is a highly sensitive spectroscopic technique used to detect and study vibrational modes of molecules adsorbed on metal or semiconducting surfaces or nanoparticles (NP) [1,2,3,4,5,6,7]. SERS intensity enhancement factors (EFs) by up to 14 orders of magnitude arise from a combination of electromagnetic (EM) and chemical (CM) mechanisms [8,9]. EM enhancement factors (~10^7^–10^8^) are dominant for metallic materials [10,11] compared with the CM enhancement (~10^3^) operating mainly among semiconductors [12,13,14,15]. Typical SERS active supports include noble metals (Au, Ag, Cu) that develop localized surface plasmon resonances (SPR) in the visible range, giving rise to the photon-vibrational coupling effects leading to SERS with EM enhancement [11,16]. In addition, if the adsorbed analyte contains a chromophore with a UV-visible absorption close to the SPR wavelength, additional enhancement can be observed due to resonance Raman (SERRS) effects [17].

Ag and Au NPs have been mainly investigated as the most highly active SERS substrates [11,18,19,20,21]. Cu NPs exhibit similar SPR properties but can show lower chemical stability toward adsorbate species [16,22,23,24,25]. Cu NPs are readily produced using solution chemistry techniques, but these approaches can lead to surface contamination due to atmospheric oxidation, as well as from the addition of surfactants such as polyethylene glycol, sodium dodecyl sulfate, etc. [26,27,28], thus hindering their use for SERS, as well as for biomedical and catalytic applications [29,30]. Laser ablation from a pure metal target in the presence of a liquid medium has been shown to provide a useful alternative to obtain SERS-active NPs [24,25,31]. Using liquids such as H_2_O or acetone results in the formation of Cu oxides [32,33,34] that reduce the SERS activity and affect the chemical stability [35]. Here, we explored the use of the oxygen-free liquids toluene (C_7_H_8_) and acetonitrile (CH_3_CN) for the Cu laser ablation experiments. The aromatic hydrocarbon toluene produced Cu NPs surrounded by an amorphous carbon matrix that resulted in quenching of the SPR. Similar results have been reported for Au NPs [36,37]. These effects could be reduced by incorporating N atoms in the ablation medium and the resulting solid matrix surrounding and supporting the Cu NPs. Previous studies have described the laser ablation of Cu and other transition metal elements using acetonitrile to obtain metal NPs deposited along with a carbon nitride support, but the nature of the support material obtained has not yet been fully established [38,39,40,41,42]. Here, we characterized the Cu NPs and the carbon nitride matrix, that we established to have a polymeric to graphitic character based on mainly sp^2^-bonded C and N species (labelled gCN for convenience), using a range of spectroscopic, diffraction, and imaging techniques. We evaluated the performance of the Cu/gCN hybrid nanomaterials for their surface-enhanced Raman or resonance Raman scattering (SERS or SERRS) performance using crystal violet (CV), methylene blue (MB), and rhodamine 6G (R6G) as probe analyte molecules adsorbed on the NP surface.

Different classes of carbon nitride materials are currently being investigated for applications including catalysis, photocatalysis, electrochemical energy storage, and conversion, and as supports for catalytically active metal NPs [43,44,45,46]. Most work to date is focused on semiconducting compounds with C_x_N_y_H_z_ compositions and polymeric to layered (“graphitic”) structures are being developed. Although these materials are often cited as graphitic (g-) C_3_N_4_, this particular stoichiometry and layered structure have only been characterized in a single study that described a triazine-based graphitic carbon nitride (TGCN), produced using the polymerization of dicyandiamide (DCDA: C_2_N_4_H_4_) in a LiCl:KCl molten salt medium [47]. That process normally results in crystalline polytriazine imide (PTI) solids containing intercalated Li^+^ and Cl^−^ ions [48]; the TGCN material consisted of dark-colored flakes formed at the molten surface or deposited on the walls of the reaction vessel. However, related studies have found that the layered gCN materials deposited from above the DCDA/molten salt mixture under analogous reaction conditions had significantly greater C:N ratios, close to 1.3 [49,50]. That composition is similar to the gCN supports formed in our laser ablation study, described below. The wide range of materials described as carbon nitrides include N-doped graphite and graphene produced by chemical or physical vapor deposition exhibit semi-metallic to semiconducting behavior, with optoelectronic properties determined by the relative C:N ratio and their distribution within the sp^2^-bonded structures [51,52,53]. Such materials have been formed via the laser ablation of graphite targets in N_2_ or NH_3_ atmospheres, or by the laser-assisted reactions of precursors in gas and liquid media, giving rise to materials containing up to ≈20 at% nitrogen in the resulting amorphous to nanocrystalline films [54,55,56,57,58,59,60]. There have been reports of embedding Cu NPs in carbon nitride materials for functional applications. Hydrothermal processing in Cu-containing ionic liquids produced a Cu/gCN electrocatalyst that showed catalytic activity for the oxygen reduction reaction (ORR) critical for fuel cell operations [61], while reacting CuCl_2_ with melamine in methanol formed supramolecular networks and carbon nitride nanosheets and nanorods with coordinated Cu^2+^ ions resulting in enhanced catalytic and visible light photocatalysis properties [62,63,64,65,66].

These last investigations indicate that electronic interactions between Cu NPs and supporting carbon nitride matrices can lead to the cooperative enhancement of catalytic and photocatalytic effects. Such interactions could be harnessed to develop other optical properties, including SERS enhancement of the vibrational signatures of molecular species adsorbed on the surface. Studies have reported combining semiconductor NPs (such as TiO_2_) with plasmonically active NPs to stabilize and enhance the performance of SERS substrates [67,68,69]. Jiang et al. described a hybrid Ag/gCN material with a stable high-performance SERS activity, demonstrating strong optoelectronic interactions that promote the SERS enhancement along with charge transfer effects that protect the Ag NPs from oxidation [70,71]. Here we applied pulsed laser ablation of a Cu target coated with acetonitrile (CH_3_CN) to create Cu/gCN hybrid nanomaterials that were characterized using a range of spectroscopic, diffraction, and imaging techniques, and we studied their SERS/SERRS activity for the probe analyte molecules crystal violet (CV), methylene blue (MB), and rhodamine 6G (R6G).

## 2. Materials and Methods

A high-purity copper plate (Cu 99.98%) with a 2-mm thickness coated with acetonitrile served as the target for the laser ablation experiments. High-purity, methylene blue (MB), crystal violet (CV) (99.5%), and rhodamine 6G (R6G) (≥95%) were purchased from Sigma Aldrich (Darmstadt, Germany) and used as SERS probe analytes. Bulk elemental analysis was carried out using an Elementar-Vario EL III system (Hanau, Germany) and X-ray photoelectron spectroscopy (XPS) was carried out using a Thermo Scientific K-Alpha instrument (Waltham Massachusetts, U.S.A) with a monochromated Al Kα source (1486.6 eV). Transmission electron microscopy (TEM) was carried out using Zeiss-EM10C-80 kV (Germany) and JEOL JEM2010-200 kV (Tokyo, Japan) instruments. The micrographs were analyzed using ImageJ software (Version 6, California, U.S.A) [72] and by manual counting to determine the mean size of NPs in the TEM images. The average Cu NP sizes were determined to be ≈6 nm, slightly lower than those estimated from Scherrer broadening of the X-ray powder diffraction data. X-ray diffraction (XRD) patterns were recorded using an X-Pert MPD (Philips, Eindhoven, Netherlands) X-ray diffractometer with Co Kα-radiation (λ = 1.79026 Å). Ultraviolet–visible (UV/vis) absorption spectra were obtained using a PG T80+ spectrophotometer (PG Instruments, Coventry, UK), Fourier Transform infrared (FTIR) spectra with a Thermo Nicolet NEXUS 670 FTIR (Waltham Massachusetts, U.S.A), and Raman spectroscopy including SERS or SERRS effects were recorded using a Thermo Nicolet Almega spectrometer (Waltham Massachusetts, U.S.A) with a 532-nm excitation provided by a Nd:YLF laser.

Pulsed laser deposition was carried out using a Ce:Nd:YAG laser (λ = 1064 nm) with a 10-ns pulse width and a 10-Hz repetition rate. The beam was focused on the Cu plate target using a 10-cm working distance lens. The target was fixed to the bottom of a glass cell and mounted on a rotator in order to avoid deep ablation. The laser pulse energy was held constant at 100 mJ/pulse. The typical diameter of the laser spot at the target was 2 mm and the acetonitrile level on the target surface was 2 mm. The pulsed laser was directed on the rotating target surface for 10 min to obtain a colloidal suspension of ablated nanomaterial particles. The suspension was then dried at 25 °C in air to provide solid powdered materials for further investigation.

To prepare SERS substrates, 1 mg of the synthesized support materials (Cu/gCN) were suspended in 5 mL of the different analyte solutions (CV, MB, and R6G) prepared with concentrations ranging between 10^−3^–10^−8^ mol/L in acetonitrile and stirred for 3 h to allow binding between the analyte and substrate. The analyte-decorated Cu/gCN solutions were centrifuged and washed multiple times with absolute acetonitrile to remove any unabsorbed analyte molecules and then the resulting powders were dispersed into 1 mL acetonitrile. SERS substrates were prepared by dropping aliquots of analyte-decorated Cu/gCN solutions (10 μL) onto a glass slide and the volatile liquid phase was left to evaporate at 25 °C in air to form a uniform coating. All Raman and SERS/SERRS experiments were carried out with the incident laser power maintained at 10 mW for a 60 µm (spot diameter) laser beam focused at different points on the sample surface maintaining a constant integration time of 100 s at a spectral resolution of 4 cm^−1^. All spectra were measured using 532-nm excitation delivered by the second harmonic of a Nd:YLF laser and were collected in the range of 400−4000 cm^−1^. All data processing and statistical analysis were carried out using the OMNIC™ software (Version 6) supplied by Thermo Nicolet (Waltham Massachusetts, U.S.A) and intensity values of the spectra were normalized according to the integration time within each data set.

## 3. Results and Discussion

### 3.1. Formation and Characterization of Cu/g-CN Materials Produced by Pulsed Laser Ablation in Acetonitrile

The interaction of high-power laser beams with a Cu target can result in several phenomena including heating, melting, and even vaporization of the surface, leading to the formation of plasma. A complex process involving adiabatic plasma expansion combined with shockwaves and the formation of cavitation bubbles result in ablation of Cu NPs that become suspended within the overlying liquid medium. The high temperatures generated by laser beam absorption using the Cu target result in bond-breaking and polymerization reactions occurring within the acetonitrile liquid phase surrounding the ablated metal NPs. Also, radiation from the laser-ablated Cu vapor plume leads to UV absorption and photolysis reactions within the acetonitrile liquid. The combination of these thermal and photolytic reaction effects, accompanied by new C–C/C–N bond formation, results in the formation of a polymerized gCN matrix that then surrounds and supports the Cu NPs formed during laser ablation treatment [38].

**Figure 1:**
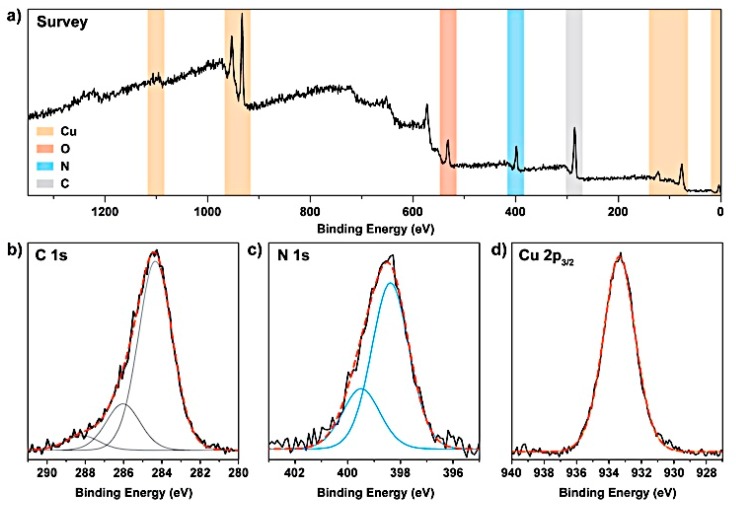
(**a**) XPS survey spectrum, (**b**) C1s, (**c**) N1s, and (**d**) Cu 2p_3/2_ XPS spectra of the Cu/gCN material produced by laser ablation under acetonitrile.

We first characterized the chemical composition of the Cu/gCN hybrid materials using bulk elemental (CNH) analysis that revealed a C:N ratio of 1.3, which was larger than those expected for g-C_3_N_4_ or polymeric to graphitic gCN materials prepared via the thermolysis of organic precursors [43,44]. However, it only slightly exceeded the C:N ratio found for layered gCN materials produced using vapor deposition above a molten salt bath from precursors such as DCDA [49,50]. Examination of the survey C1s and N1s spectra obtained from XPS measurements confirms this observation (Figure 1a–c). The C1s spectra could be fit using three mixed Gaussian–Lorentzian (GL) peaks at 284.3, 286.0, and 288.2 eV. The peak at 288.2 eV appears at a position that is characteristic of sp^2^ C atoms bonded within a cyclic triazine or heptazine systems [44], although this constituted only a minor (5.5%) component of the overall C contribution. The peak at 286.0 eV (18.55% of the C contribution) was assigned to C–N bonded species, although it might also contain a contribution from C–O bonded species that are often recorded for gCN materials due to surface oxidation [44,50]. It is probable that the remainder of the C–N bonding was represented by a significant, but unresolvable, contribution to the peak at 284.3 eV, along with a component from adventitious carbon [50,53]. The presence of the C1s peak at 286.0 eV, as well as a significant proportion of that observed at 284.3 eV, confirmed the presence of significant C–N interactions within the sample. The N1s spectrum (Figure 1c) was best fit with two GL peaks, one at 398.4 eV, and a second contribution at 399.5 eV. The smaller peak resembles that observed for melamine and is usually interpreted to be indicative of C–N–H uncondensed amino (–NH_2_) groups [44]. We note that the IR spectra discussed below indicate the presence of N–H bonded species. The peak at the higher binding energy (BE) resulted from the remaining C–N species, which included C–N=C bonded units indicated by the peak in the C1s spectrum. The IR and Raman data also indicated the presence of terminal nitrile (–C≡N) units that contributed to the N1s signal. The C1s:N1s peak ratio found from the XPS data was 4.76. This was significantly larger than the value determined using bulk elemental analysis (1.33), but it was certainly affected by a significant contribution from adventitious carbon. However, the fact that the C:N ratio was substantially greater than 1 clearly indicates that both C–C and C–N species were present within the gCN materials. The Cu 2p_3/2_ peak (Figure 1d) exhibits a single sharp feature that could be fitted using a GL peak at 933.3 eV. The shape and position of this feature, as well as the absence of higher BE satellites, rule out the presence of Cu(II), but Cu(I) and Cu(0) species both have similar XPS signatures, and it is possible that either or both of these species might be present within the sample [73].

**Figure 2:**
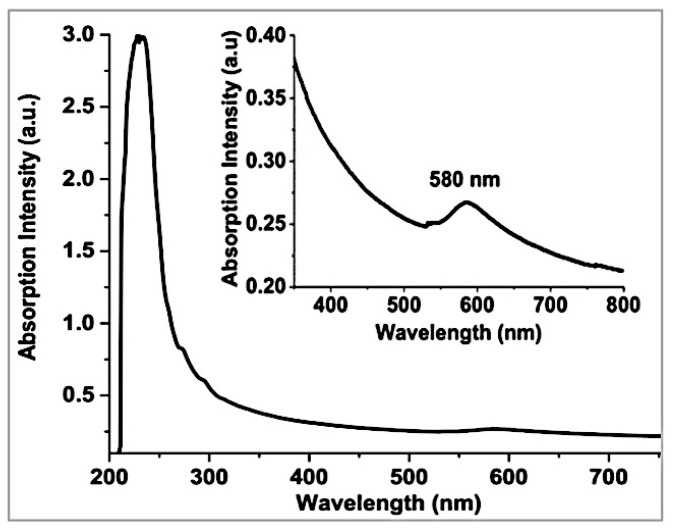
UV–visible absorption spectrum of a typical Cu/gCN nanomaterial synthesized using pulsed laser ablation in acetonitrile. The inset shows a vertically expanded region of the spectrum around the position of the surface plasmon resonance for Cu NPs.

UV-visible absorption spectra of the Cu/gCN nanomaterials obtained in suspension in acetonitrile following the laser ablation synthesis exhibited a peak at ≈580 nm due to the localized surface plasmon resonance (SPR) band of the Cu NPs (Figure 2) [16,24,74].

**Figure 3:**
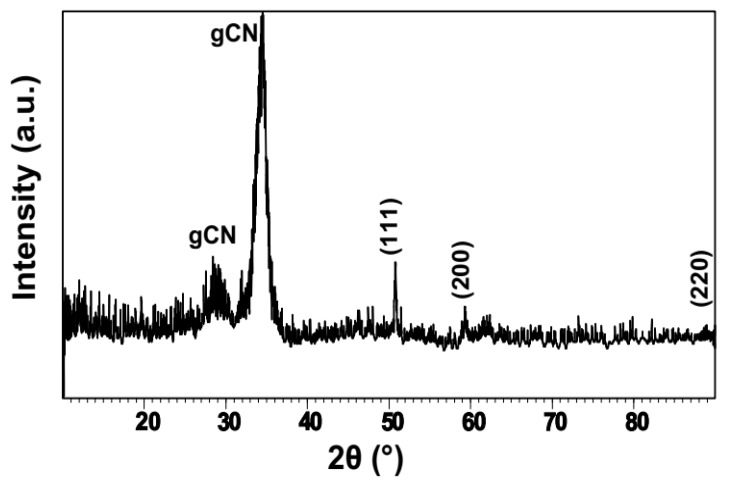
Powder XRD pattern (obtained using Co Kα radiation, λ = 1.79026 Å) of the Cu/gCN material formed using pulsed laser ablation in acetonitrile. The weak sharp peaks due to crystalline Cu NPs are labeled with their {hkl} values. The strong broad features are due to the gCN support generated during the laser ablation synthesis from acetonitrile.

The XRD pattern of dried powders obtained using Co Kα radiation showed weak sharp peaks that were distinct from the broad and more intense features from the amorphous gCN matrix (Figure 3). The weak peaks occurring at 50.7° and 59.2° 2θ (λ = 1.79026 Å) were identified with the {111} and {200} reflections of crystalline Cu (2.09 and 1.08 Å, respectively). A very weak feature could just be distinguished at 89.1° 2θ (1.27 Å) that corresponded to the {220} reflection. Examining the widths of these features indicated an NP size with an average mean diameter ≈32 nm estimated using Scherrer’s formula (Table 1). The pattern was dominated by a strong, broad peak at 34.3° 2θ (3.03 Å), along with a weaker feature at 28.9° 2θ (3.59 Å). These resembled the broad reflections observed for polymeric to graphitic carbon nitride materials prepared using thermolysis of organic precursors, where they are commonly interpreted in relation to the {002} and {100} planes of polytriazine to polyheptazine graphitic layered structures, even though the actual materials are known to be incompletely polymerized and contain ribbon-like units along with partially formed sheets [44,75,76,77]. Similar features were also found for nanocrystalline layered materials with higher C:N ratios close to those observed for the gCN supports created in this study [44,49,50]. We note, however, that the main XRD peak occurred at a value that is ≈11% smaller than the interplanar *d*_002_ spacing of crystalline graphite (*d*_002_ = 3.28 Å), or gCN compounds with both low (~3.26 Å) and high C:N ratios (~3.24 Å) [44,49,50,75,76,77].

**Figure 4:**
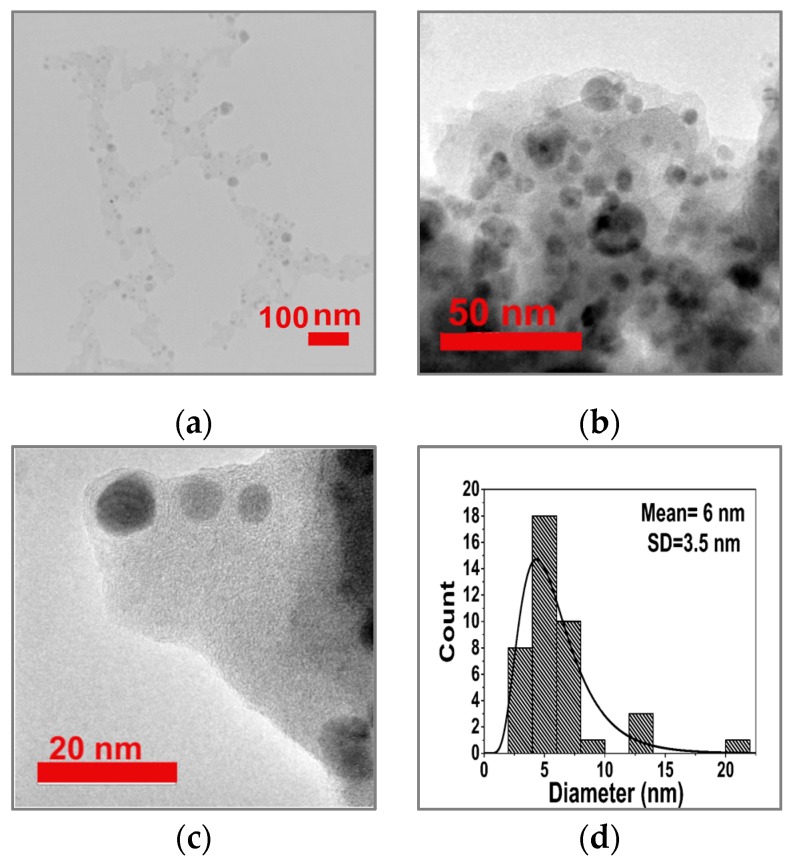
TEM images of Cu NPs embedded in the amorphous gCN support. (**a**) Low-resolution image obtained using a Zeiss-EM10C-80 kV instrument; (**b**,**c**) High-resolution images obtained using a JEOL JEM2010-200 kV TEM. (**d**) Mean size (nm) and distribution by analysis of ≈40 Cu NPs.

Cu NPs were shown to be distributed throughout the gCN matrix via TEM imaging (Figure 4a–d). By counting ≈40 particles, the mean diameter was found to be ≈6 nm by analyzing the images using ImageJ software [72]. A few particles in the field of view were observed to have diameters in the 10–25 nm range. The greater average particle size (≈30 nm) indicated using the XRD results could have been the result of the presence of large NPs within the bulk materials that were not sampled in the TEM experiments, although Scherrer analysis of the X-ray peaks is not always reliable. The gCN supporting matrix revealed a turbostratic texture with an "onion-like" structure developed around the Cu NPs (Figure 4c).

**Figure 5:**
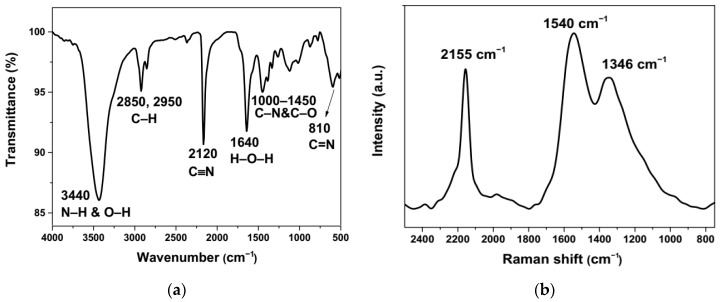
(**a**) FTIR and (**b**) Raman spectra of Cu/gCN hybrid material synthesized using pulsed laser ablation in acetonitrile.

FTIR spectra of a pressed KBr disc of the powdered Cu/gCN sample contained a strong band at 3340 cm^−1^ due to N–H stretching from the gCN material, combined with a contribution from O–H stretching from surface-adsorbed H_2_O (Figure 5a) [78,79]. The peak at 1640 cm^−1^ was due to molecular H_2_O bending vibrations [78]. The features at 2850–2950 cm^−1^ indicated the presence of sp^3^-hybridized C–H bonds [80]. Features in the 1000–1450 cm^−1^ region can be assigned to C–N and possibly C–O stretching vibrations [44,50]. The sharp peak at 2120 cm^−1^ was due to terminal C≡N bond stretching within the incompletely condensed carbon nitride matrix [79,80]. The strong Raman peak at 2155 cm^−1^ confirmed this assignment (Figure 5b), although it is not clear why there should be a 35 cm^−1^ difference between the IR and Raman data. This could indicate a vibrational interaction between adjacent nitrile groups within the structure giving rise to symmetric and antisymmetric combinations; however, typically the IR mode should occur at a higher wavenumber. Our observation of this feature clearly indicates that the gCN material produced by laser ablation from acetonitrile was not fully polymerized into graphitic sheets. However, the sharp peak in the IR spectrum near 810 cm^−1^ was typical of breathing modes of ring units in carbonaceous and C*_x_*N*_y_* materials. Also, the Raman spectrum contained two main bands at 1346 and 1540 cm^−1^ that resembled the “G” and “D” bands of disordered graphite, amorphous carbon, and graphitic carbon nitride materials, associated with C–N/C–C stretching vibrations within the gCN matrix [44,50,79,80].

### 3.2. SERS/SERRS Activity of Analytes on Cu/gCN Hybrid Support Produced Using Pulsed Laser Deposition

**Figure 6:**
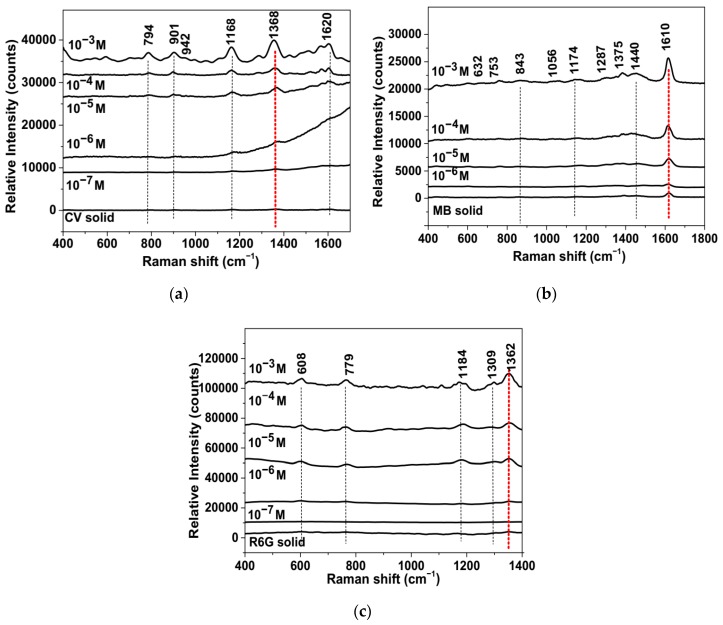
Raman spectra of (**a**) CV, (**b**) MB, and (**c**) R6G powders compared with SERS/SERRS spectra of the analytes with different concentrations decorated on a Cu/g-CN substrate. All spectra were plotted following baseline subtraction (see Figure S1). The red line indicates the characteristic peak used to determine the SERS activity (see text and Supporting Information). The spectra have been vertically displaced for clarity. The counts shown correspond to an integration time of 100 s of 50 accumulated scans, each with a 2-s exposure period. We note that for the CV sample prepared with a 10^−7^ M concentration, an additional fluorescence background contribution remained that was not fully accounted for by the baseline subtraction procedure. This was most likely due to some analyte molecules that were not completely removed from the underlying glass support by the washing procedure.

We tested the SERS/SERRS activity of the CuNP/g-CN hybrid using the dye molecules CV, MB, and R6G as analyte probes. A drop of each the analyte-decorated Cu/gCN solutions prepared with various due concentrations (10^−3^–10^−7^ M) was placed on the prepared glass slide substrates separately and allowed to dry before recording surface-enhanced spectra. “Normal” and SERS/SERRS spectra are compared in Figure 6, after subtraction of the background fluorescence (Figure S1). No additional bands were observed from the gCN support material (Figure S2). All spectra were obtained using a total integration time of 100 s from 50 accumulated scans with a 2-s exposure. The spectra shown in Figure 6 have been displaced vertically to more clearly show the SERS/SERRS enhancement.

Because CV has a main UV-visible absorption band extending between 420–640 nm with its principal maximum near 580 nm, resonance Raman (RR) effects were expected to occur for the bulk crystalline sample using 532 nm excitation [81]. However, using the acquisition conditions applied here, we could barely observe the vibrational peaks of CV rising above the background when presented at the same vertical expansion as the SERS/SERRS spectra (Figure 6a). For the analyte deposited on the Cu/gCN support at 10^−3^ M concentration, we clearly observed the spectrum of CV with peaks at 794, 901, 942, 1168, 1368, and 1620 cm^−1^ (Figure 6a). Although the positions and relative intensities generally resembled those of published spectra of the solid sample obtained with different laser wavelengths [81], we observed the most significant enhancement in the intensity of the feature at 1368 cm^−1^ that contains a main contribution from symmetric *ϕ* –N (*ϕ* = phenyl) stretching [81]. As the concentration of adsorbed CV molecules was reduced by decadic amounts, the main peaks could still be clearly distinguished from the background down to 10^−7^ M (Figure 6a).

MB exhibited a UV-visible absorption that had barely begun at the 532 nm laser wavelength, and so it was expected that any RR or pre-resonance effects would be minimal for the bulk sample, while the background fluorescence should be low [82]. That was consistent with our data for the solid sample, for which the principal peak recorded at ≈1610 cm^−1^ in our study was clearly observed for the counting conditions used here (Figure 6b). However, the Cu NP SPR occurred at 580 nm, which lay within the electronic absorption profile of the chromophore, and so the SERS enhancement effects could also include SERRS. We observed SERS-active Raman peaks at 632, 753, 843, 1056, 1174, 1287, 1375, 1440, and 1610 cm^−1^ for our sample prepared from a 10^−3^ M analyte solution, with a relative intensity pattern that resembled that obtained by Anastasopolous et al. for MB molecules at 10^−4^ M concentration adsorbed on Ag NPs and excited using a 514.5 nm excitation [82]. We can clearly observe the dominant 1610 cm^−1^ peak down to a 10^−6^ M concentration, with a relative intensity that was only slightly lower than that for the bulk solid (Figure 6b).

R6G exhibited an electronic absorption maximum at 530 nm that matched the laser excitation wavelength and also lay close to the SPR of Cu NPs, and so RR effects were expected to be observed for both the bulk solid and adsorbed molecules in our study using a 532-nm laser excitation. As observed by previous researchers, the SERRS effect on Au nanoparticles or roughened Cu surfaces resulted in the appearance of the characteristic Raman peaks of the adsorbed molecules using red (632.8 nm) to blue (488 nm) excitation lines [83,84]. Our data show that the spectrum could be detected down to at least 10^−6^ M, where the relative intensities were comparable to those of the bulk solid (Figure 6c). Overall, our results demonstrated that the Cu/g-CN hybrid substrate exhibited good SERS/SERRS sensitivity for the CV, MB, and R6G molecular analytes.

### 3.3. Estimation of SERS/SERRS Enhancement Factors

**Figure 7:**
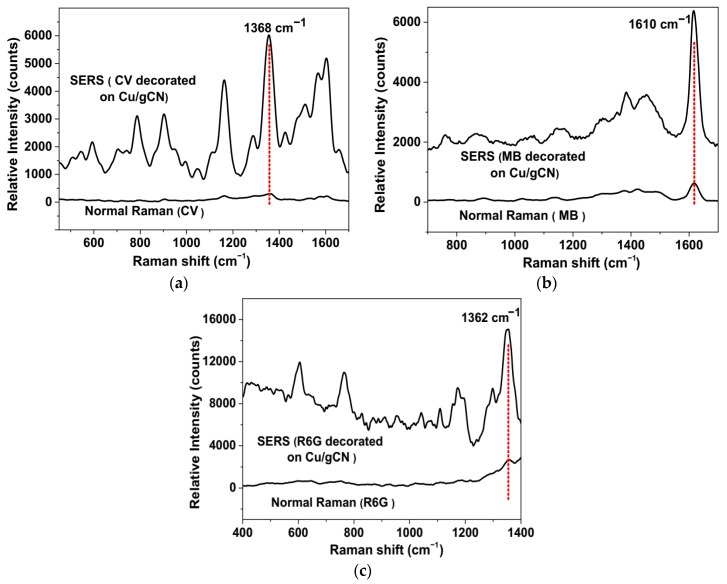
Comparison of the “normal” (i.e., non-SERS) Raman spectra of (**a**) CV, (**b**) MB, and (**c**) R6G with the SERS/SERRS spectra of the three analytes decorated on a Cu/gCN support. The concentration of the analytes was 10^−3^ M for recording both the “normal” Raman and SERS spectra. Spectra were plotted following subtraction of the fluorescence background from the organic molecules (see Figure S1). The red line indicates the characteristic peak used to determine the SERS activity (see text and Supporting Information). The spectra have been vertically displaced for clarity. The counts represent an integration time of 100 s of 50 accumulated scans, each with a 2 s exposure period in all cases.

In order to further investigate the SERS/SERRS enhancement activities of the Cu/gCN substrate, the surface was decorated with a sufficiently high concentration of analyte molecules (CV, MB, and R6G, 10^−3^ M) to ensure full a monolayer coverage of the NP surface. We assumed that the probe molecules were uniformly adsorbed on the support surfaces. The relative intensities of characteristic peaks for the three molecules were then compared with those obtained from a dried spot (2 μL; ≈10 mm^2^) of pure analyte solution prepared at the same concentration (Figure 7a–c). The peaks at 1368, 1610, and 1362 cm^−1^ were considered for CV, MB, and R6G analytes, respectively. SERS/SERRS enhancement factors (*EF*s) were then estimated using the following equation [85,86,87]:

(1) EF =(ISERSnSERS)(IRamannRaman)

Here, *n_Raman_* and *n_SERS_* were the number of analyte molecules included within the laser beam sampling volume for the normal Raman and SERS samples, respectively. *I_SERS_* and *I_Raman_* were the absolute intensity values measured for the same mode in the Raman and SERS/SERRS spectra of the analyte. The area of the laser illumination (*A_laser_*) constituted a circle ≈30 µm in radius (*r_laser_*). The focused laser beam penetrated ≈4 µm into the material [86,88]. We considered the Cu NPs to be spherical with a radius (*r_NP_*) of ≈16 nm (estimated from the XRD and TEM results), and that the particles were half-way embedded into the gCN matrix. We neglected spaces between the NPs. The number of analyte molecules illuminated by the laser beam for the solid and SERS samples were then obtained using:

(2) nSERS =(nNPs .ANP)(AAnalyte)

Here, *n_NPs_*, *A_NP_*, and *A_Analyte_* were the number of NPs in the area illuminated by the laser, the surface area per NP, and the analyte molecule surface area, respectively. *n_Raman_* was then obtained using the following relation:

(3) nRaman =(NA .VAnalyte .dAnalyte)(MAnalyte)

where *N_A_* is Avogadro’s number, and *V_Analyte_*, *d_Analyte_*, and *M_Analyte_* were the effective volume of analyte in the laser beam, the density, and molecular weights of the related analyte (Table 2), respectively. The *EF*s were then estimated using Equation (1) for the different analytes (Table 2). The determined values were ≈7.2 × 10^7^, 2.3 × 10^7^, and 1.3 × 10^7^ for the CV, MB, and R6G analytes on the Cu/gCN substrate, respectively (see Method S1 for further details).

Because of the several assumptions and approximations used to obtain these *EF*s, they can only be considered as guide values for comparison with SERS and SERRS *EF* values recorded for these and related analytes on noble metal nanoparticles supported on semiconducting surfaces. Su et al. prepared a Au/MoS_2_ nanocomposite using in situ growth of Au NPs on MoS_2_ nanosheet surfaces, and demonstrated that the Au@MoS_2_ substrates exhibited an 8.2 × 10^5^ enhancement for the 1362 cm^−1^ Raman mode of R6G [89]. Jiang et al. reported that g-C_3_N_4_/Ag NPs exhibited *EF*s of 4.6 × 10^8^ and 2.1 × 10^9^ using CV as an analyte [70,71]. These authors linked the large *EF* values to multiple enhancement contributions, and that charge transfer between the g-C_3_N_4_ and the metal surface protected the Ag NPs from oxidation. There are only a few literature reports on the use of pure Cu NPs as a SERS substrate, either supported or in colloidal suspension [16,23,24,25]. Dendisová-Vyškovská et al. found *EF* = 1.7 × 10^5^ for the 1590 cm^−1^ band of 4-aminobenzenethiol (4-ABT) [16], and Ding et al. obtained an *EF* = 1.6 × 10^7^ for CV adsorbed on an array of Cu NPs deposited using sputtering on the surface of a monolayered colloidal crystal of 350 nm polystyrene spheres [23]. The SERS/SERRS results varied dramatically as a function of NP size controlled by the deposition time, with the most intense spectra recorded for an 18 min deposition with particles ≈150–200 nm in size. Although the exciting wavelength (532 nm) was the same as that used in our study, the SERS/SERRS spectrum recorded for CV was slightly different to our result in that the 1619 cm^−1^ band was much more intense. However, the fact that the *EF*s obtained in both studies is comparable is encouraging.

## 4. Conclusions

Our results confirm that Cu/gCN nanomaterial composites can be useful for the development of SERS/SERRS applications. Our Cu NPs on sp^2^-bonded carbon nitride supports were fabricated using pulsed laser ablation of a Cu plate immersed in acetonitrile solvent. We carried out chemical, structural, and spectroscopic analyses to study the nature of the Cu NPs and their gCN support. The N:C ratio of the gCN material determined using bulk analysis was ≈1.3, significantly lower than the semiconducting carbon nitride compounds based on heptazine- to triazine-based structures, but it contained close-to-layered gCN materials containing mixed C–N/C–C bonding prepared using vapor deposition techniques. Such materials might contain locally electronically conducting domains. The degree of layer polymerization was incomplete, as shown by strong IR and Raman signals from terminal C≡N bonds. The Cu/gCN hybrid nanocomposites exhibited strong visible absorption extending toward the near-IR region with an SPR signal from the metal NPs at 580 nm. SERS/SERRS enhancement activity was tested using three analyte molecules (CV, MB, and R6G) prepared with initial solution concentrations of 10^−3^–10^−7^ mol/L in acetonitrile and adsorbed onto the Cu/gCN nanocomposite surfaces. The observed SERS/SERRS enhancement factors were on the order of 10^7^, comparable with *EF* values found for nanoparticle arrays of Cu produced using sputtering onto templates and other noble metal NPs supported by gCN materials. Further studies are now needed to investigate the possibility of cooperative interactions between the gCN support and the metal NPs that could enhance the optoelectronic effects leading to SERS/SERRS enhancement, as well as in stabilizing the Cu NPs. 

## Figures and Tables

**Table 1:** Summary of X-ray diffraction data obtained using Co Kα radiation (λ = 1.79026 Å) for a typical Cu/gCN material prepared using pulsed laser ablation in acetonitrile. * The assignment of peaks for gCN is discussed in the text.

Angle (2θ)	{hkl}	FWHM (2θ)	d-spacing (Å)	Height (counts)	Identification
28.9	*	3.3	3.59	27.2	gCN
34.3	*	1.6	3.03	153.9	gCN
50.7	{111}	0.3	2.09	36.9	Cu
59.2	{200}	0.4	1.08	14.6	Cu
89.1	{220}	3.2	1.27	5.4	Cu

**Table 2:** Physical characteristics of the analytes used to estimate *EF*s.

Analyte	Density (g/mL)	Surface Area (nm^2^)	*n_Raman_*/*n_SERS_*	*I_SERS_*/*I_Raman_*	Cu/g-CN *EF*s
Crystal violet	1	1.2	5.4 × 10^15^π/1.5 × 10^9^π	20	7.2 × 10^7^
Methylene blue	0.98	1.3	6.6 × 10^15^π/1.4 × 10^9^π	6.6	2.3 × 10^7^
Rhodamine 6G	1.26	0.6	6 × 10^15^π/3 × 10^9^π	6.4	1.3 × 10^7^

## Supplementary Materials

The Supplementary Materials are available online at https://www.mdpi.com/2079-4991/9/9/1223/s1.
