# Supplementary material for: SERS-Active Cu Nanoparticles on Carbon Nitride Support Fabricated Using Pulsed Laser Ablation
Source: Nanomaterials (Basel). 2019 Aug 29;9(9):1223. doi: 10.3390/nano9091223 (PMC6780541; doi:10.3390/nano9091223)
Supplement: Supplementary file 1 [file nanomaterials-09-01223-s001.pdf]

Supporting Information

SERS-Active Cu Nanoparticles on Carbon Nitride Support Fabricated Using Pulsed Laser Ablation

Hossein Dizajghorbani-Aghdam [1], Thomas S. Miller [2], Rasoul Malekfar [1,*] and Paul F. McMillan [3,*]

Method S1 : Details of the EF calculation

In order to investigate the SERS/SERRS enhancement due to the Cu/gCN substrate, a sufficiently high concentration of analyte molecules (CV, MB, and R6G; 10^{-3} M) was deposited on the support to ensure a full monolayer coverage of the NP surface. The data were compared with "normal" (i.e., non-SERS) spectra obtained for a dried spot (2 µl ~ 10 mm^2) of the solid sample prepared from a 10^{-3} M concentration of each analyte solution (Figure 7a–c in the main text). We considered the intensity of the characteristic 1368 cm^{-1} peak for CV, the 1610 cm^{-1} peak for MB, and the 1362 cm^{-1} peak for R6G, to estimate the SERS enhancement factors (EF) in the SERS spectra for the analyte molecules deposited on the Cu NP/gCN films. A hybrid model was adopted to obtain the EFs [1–4]. However, we are well aware that this method of estimating EF can be associated with several problems that we have tried to account for in our treatment [1].

$$\text{EF} = \frac{I\ SERS / n\ SERS}{I\ Raman / n\ Raman}$$

The area of the laser illumination (A_{laser}) at the sample surface describes a circle ≈30 µm in radius (r_{laser}). The Cu NPs were spherical with a radius (r_{NP}) of ≈16 nm (obtained from XRD and TEM results), the NPs were considered to be approximately half-way embedded into the gCN matrix, and spaces between NPs are neglected. The penetration depth of the focused laser into the overall material was estimated as being ≈4 µm. These assumptions must obviously be considered as only representing a first approximation to the true situation. We realize that our calculations can only be considered as a developing guide values for comparing EF values for similar substrates.

The following physical parameters were used to calculate n_{Raman} and n_{SERS} for the CV sample [5]: density (d_{cv}) = 1 g/mL, molecular weight (M_{CV}) = 407.979 g/mol, and surface area (A_{cv}) = 1.2 nm^2. A_{laser} was then obtained using:

$$A_{laser} = \pi r^2_{laser}$$
$$= 9\pi \times 10^8\ (\text{nm}^2)$$

The number of NPs (n_{NPs}) in the area of the laser illumination was then:

$$n_{NPs} = A_{laser}/A_{NP,cross\text{-}section}$$
$$= 3.4 \times 10^5$$

The surface area for each NP (A_{NP}) was estimated using:

$$A_{NP} = 2\pi r^2_{NP}$$
$$= 5312\pi\ (\text{nm}^2)$$

so that the total surface area of NPs in the laser beam ($A_{plasmonic}$) was:

$$A_{plasmonic} = n_{NPs} \times A_{NP}$$
$$= 1.8 \times 10^9\pi\ (nm^2)$$

and n_{SERS} was given using:

$$n_{SERS} = A_{plasmonic}/A_{cv}$$
$$= 1.5 \times 10^9\pi$$

The effective volume of crystal violet (V_{cv}) in the laser beam was expressed as:

$$V_{CV} = \pi r^2_{laser}h \text{ (penetration depth of the focused laser: 4 μm)}$$
$$= 3.6 \times 10^{15}\pi\ (nm^3)$$

The moles (n_{cv}) of crystal violet present within this volume was:

$$n_{cv} = V_{cv}d_{cv}/M_{cv}$$
$$= 9 \times 10^{-9}\pi\ (mol)$$

For the "normal" Raman spectrum of the powdered sample, n_{Raman} was calculated as:

$$n_{Raman} = N_A \times n_{cv}$$
$$= 5.4\times 10^{15}\ \pi$$

Therefore, for the CV analyte, $n_{SERS} = 1.5 \times 10^9\pi$ and $n_{Raman} = 5.4 \times 10^{15}\pi$. The intensities recorded for the characteristic (non-resonant) Raman peaks observed in SERS and the "normal" Raman spectrum of the powdered sample, I_{SERS} and I_{Raman}, were 5200 and 260, respectively.

Thus, the final EF was:

$$EF = \frac{I\ SERS/n\ SERS}{I\ Raman/n\ Raman} = 20 \times 3.6 \times 10^6 = 7.2 \times 10^7$$

We performed a similar calculation for MB, using the following data:

Density of MB (d_{MB}) = 0.98 g/mL, molecular weight of MB (M_{MB}) = 319 g/mol, and surface area (A_{MB}) = 1.3 nm^2. As above:

$$n_{SERS} = 1.4 \times 10^9\pi \text{ and } n_{Raman} = 6.6 \times 10^{15}\pi$$

The intensities of the SERS and Raman peaks (I_{SERS} and I_{Raman}) were 4300 and 650, respectively, leading to an EF value of:

$$EF = \frac{I\ SERS/n\ SERS}{I\ Raman/n\ Raman} = 6.6 \times 3.6 \times 10^6 = 2.3 \times 10^7$$

The same calculation for R6G was carried out using: density of R6G (d_{R6G}) 1.26 g/mL, molecular weight (M_{R6G}) 442 g/mol, and surface area (A_{R6G}) = 0.6 nm^2 gave:

$$n_{SERS} = 3 \times 10^9\pi \text{ and } n_{Raman} = 6 \times 10^{15}\pi$$

The intensities of the characteristic peaks (I_{SERS} and I_{Raman}) were 9000 and 1400, respectively, leading to an EF value of:

$$\text{EF} = \frac{I\,SERS/_{n\,SERS}}{I\,Raman/_{n\,Raman}} = 6.4 \times 2 \times 10^{6} = 1.3 \times 10^{7}$$

Method S2: Fluorescence baseline subtraction of Raman and SERS spectra. The Raman and SERS spectra excited by the 532-nm laser exhibited a strong fluorescence background from each of the molecular analyte compounds. This was subtracted using a baseline correction procedure implemented using the OMNIC™ software, illustrated below for the R6G bulk solid powdered sample.

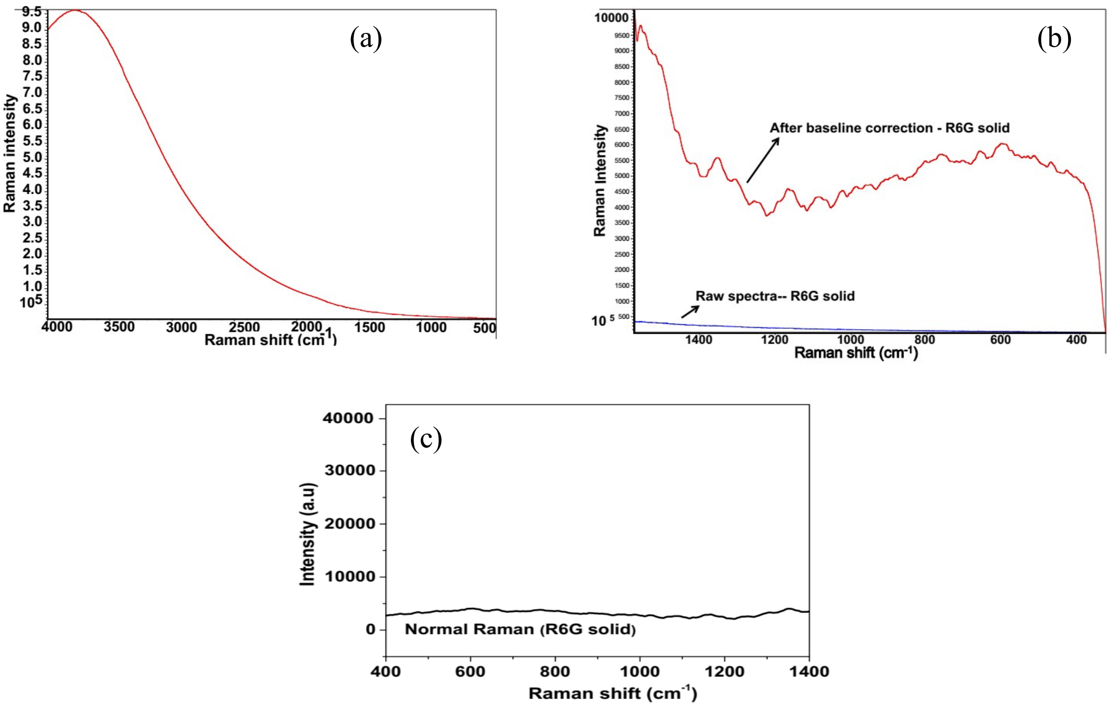

Figure S1: (**a**) The Raman spectrum obtained for solid powdered R6G excited using 532-nm laser radiation. The signal was dominated by strong fluorescence from the molecular species that obscured the "normal" (i.e., non-SERS) Raman peaks. (**b**) These peaks were only observed following baseline correction, and the final spectrum (**c**) plotted as Figure 6c in the main text included a further correction to provide a flat baseline for ready comparison between the "normal" Raman and SERS spectra. We note that Figure S1 (a & b) has the relative cm^{-1} scale reversed to correspond to the data shown in the main text (Figure 6c).

Method S3: The Raman intensity of the Cu-gCN substrate bands located at 1346 and 1540 cm^{-1} was even lower than the overlapped SERS bands (at 1368 and 1620 cm^{-1}) for CV with 10^{-7} M concentration decorated on Cu-gCN. Therefore, the bands of the substrate were not visible on the SERS spectra (see Figure 6 in the main text).

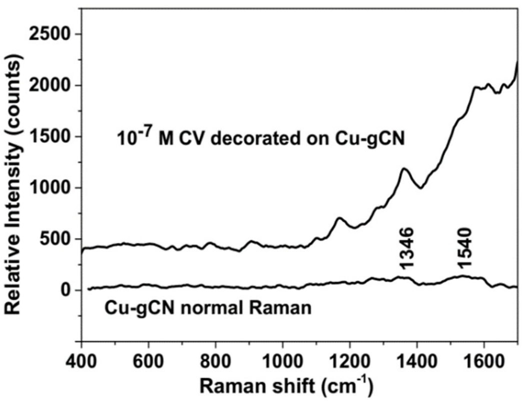

Figure S2: Comparison of the "normal" Raman spectrum of Cu/gCN substrate with a SERS/SERRS spectrum of 10^{-7} M concentration CV-decorated analytes on the same Cu/gCN substrate. Spectra were plotted after subtraction of the fluorescence background from the organic molecule (see Figure S1). The spectra have been displaced vertically for ease of comparison. The counts represent an integration time of 100 seconds of 50 accumulated scans, each with a 2-s exposure period for both samples.

References

1. Ru, E. Le; Blackie, E.; Meyer, M.; Etchegion, P.. Surface enhanced Raman scattering enhancement factors: a comprehensive study. *J. Phys. Chem. C.* **2007**, *111*, 13794–13803.

2. Jiang, L.; You, T.; Yin, P.; Shang, Y.; Zhang, D.; Guo, L.; Yang, S. Surface-enhanced Raman scattering spectra of adsorbates on Cu2O nanospheres: Charge-transfer and electromagnetic enhancement. *Nanoscale.* **2013**, *5*, 2784–2789.

3. He, S.; Chua, J.; Tan, E.K.M.; Kah, J.C.Y. Optimizing the SERS enhancement of a facile gold nanostar immobilized paper-based SERS substrate. *RSC Adv.* **2017**, *7*, 16264–16272.

4. Khlebtsov, B.N.; Khanadeev, V.A.; Panfilova, E. V.; Bratashov, D.N.; Khlebtsov, N.G. Gold nanoisland films as reproducible SERS substrates for highly sensitive detection of fungicides. *ACS Appl. Mater. Interfaces* **2015**, *7*, 6518–6529.

5. Xu, Y.; Konrad, M.P.; Trotter, J.L.; Mccoy, C.P.; Bell, S.E.J. Rapid one-pot preparation of large freestanding nanoparticle-polymer films. *Small* **2016**, *13*, 1–6.
